# Addressing the unmet challenge of pain in rare bone diseases: new insights from the RUDY UK registry

**DOI:** 10.1186/s13023-025-04167-4

**Published:** 2026-01-29

**Authors:** Melanie Alice Legrand, Franz Aaron Clemeno, Roland Chapurlat, Anushka Irani, Muhammad Kassim Javaid

**Affiliations:** 1 Botnar Research Centre, Nuffield Department of Orthopaedics, Rheumatology and Musculoskeletal Sciences, University of Oxford, Oxford, UK; 2 French Reference Center for Fibrous Dysplasia, Rheumatology Department, Hospital E.Herriot, Hospices Civils de Lyon, University of Lyon 1, Lyon, France; 3 Division of Rheumatology, Mayo Clinic, Florida, USA

**Keywords:** Rare bone disease, Fibrous Dysplasia/McCune-Albright, Osteogenesis imperfecta, X-linked hypophosphatemia, Bone pain, Pain phenotype

## Abstract

**Background:**

Pain is a common symptom in many rare bone disorders, often linked to depression and a substantial decline in quality of life. However, there is little information on the quality of the pain which may provide insights into pain mechanisms. This study aimed to describe and compare the frequency and characteristics of self-reported pain in adults with Fibrous Dysplasia of Bone/McCune-Albright Syndrome (FD/MAS), Osteogenesis Imperfecta (OI), and X-linked Hypophosphatemia (XLH).

**Methods:**

A cross-sectional study was conducted using the online UK RUDY registry. Adults with self -reported FD/MAS, OI, and XLH who completed the painDETECT questionnaire (PD-Q) were included. Pain prevalence and phenotypes were assessed using baseline PD-Q responses which were also mapped to a modified widespread pain index as a measure of generalized pain. Descriptive analyses were performed using R^®^.

**Results:**

A total of 281 adults completed the baseline PD-Q (94 FD/MAS, 94 OI, and 93 XLH). Among these, 86% of patients currently experienced pain and 47% reported severe strongest pain in the past four weeks, with no significant differences between conditions. Pain prevalence and phenotype were similar across diseases, though pain sites differed. Neuropathic-like pain and female sex were significantly associated with poorer pain outcomes, including higher pain prevalence and intensity (*p* < 0.05). Generalized pain (18%) was significantly associated with moderate to severe anxiety (*p* = 0.03), depression (*p* < 0.001) and sleep impairment (*p* < 0.001).

**Conclusion:**

Despite distinct pathophysiological mechanisms, pain distribution appears similar across these bone diseases, suggesting a major role for non-skeletal factors. Generalized pain was frequent and associated with anxiety, depression, and sleep disturbances, suggesting nociplastic features maybe a significant driver of pain in adults with rare bone diseases.

**Supplementary Information:**

The online version contains supplementary material available at 10.1186/s13023-025-04167-4.

## Background

Pain is a common and debilitating symptom in patients with bone disorders [1], including rare bone diseases, and is frequently associated with depression and a marked reduction in quality of life [2, 3].

In rare bone diseases, pain remains poorly understood, with little information on its underlying mechanisms and the qualitative aspects of the pain experienced. Pain management in this population is particularly challenging, due to the lack of evidence-based treatment strategies combined with limited therapeutic options. Currently, treatment decisions are often based on physician experience and expert consensus, with the efficacy of many pain-relieving medications remaining uncertain due to few randomized controlled trials in this patient population. This knowledge gap has resulted in insufficient pain management strategies, leaving many patients with inadequate relief.

Bone pain is multifactorial, driven by both bone-related factors and extra-osseous factors (e.g., demographic characteristics, comorbidities and psychosocial or emotional factors), and is believed to be associated with complex pathological processes such as fractures, bone deformities, or alterations in bone mineralization [4, 5]. Identifying the contributing factors that influence pain severity and distribution could improve our understanding of the mechanisms driving pain and to optimize pain management, ultimately improving patient outcomes in rare bone diseases.

Pain is a complex and subjective experience, which makes it challenging to capture comprehensively. Studies often focus only on current pain intensity, but other essential aspects and characteristics, such as pain quality or the fluctuation over time, are frequently neglected. In this context, online registries, provide valuable tools for assessing multidimensional aspects of pain and are particularly useful in rare diseases, enabling large-size sample data collection despite the rarity of the condition.

To address this, we conducted a cross-sectional study to explore pain experiences and identify the potential contributing factors across a range of rare bone diseases in adults, using the UK RUDY registry.

## Methods

### Design and data source

A cross-sectional study was conducted using the online RUDY registry. All participants in RUDY provided informed consent for the collection and use of their data for research (RUDY LREC 14/SC/0126 & 17/SC/0501). Adults with self-reported FD/MAS, OI, or XLH registered in the UK-based RUDY registry who had completed the painDETECT questionnaire (PD-Q) at baseline by -january 6th, 2025 were included. Demographic data, including age and sex, disease-specific characteristics (monostotic or polyostotic form for FD, OI type), and questionnaire data capturing pain and quality of life measures were collected from RUDY at baseline.

### Outcomes measures

**Fig. 1:**
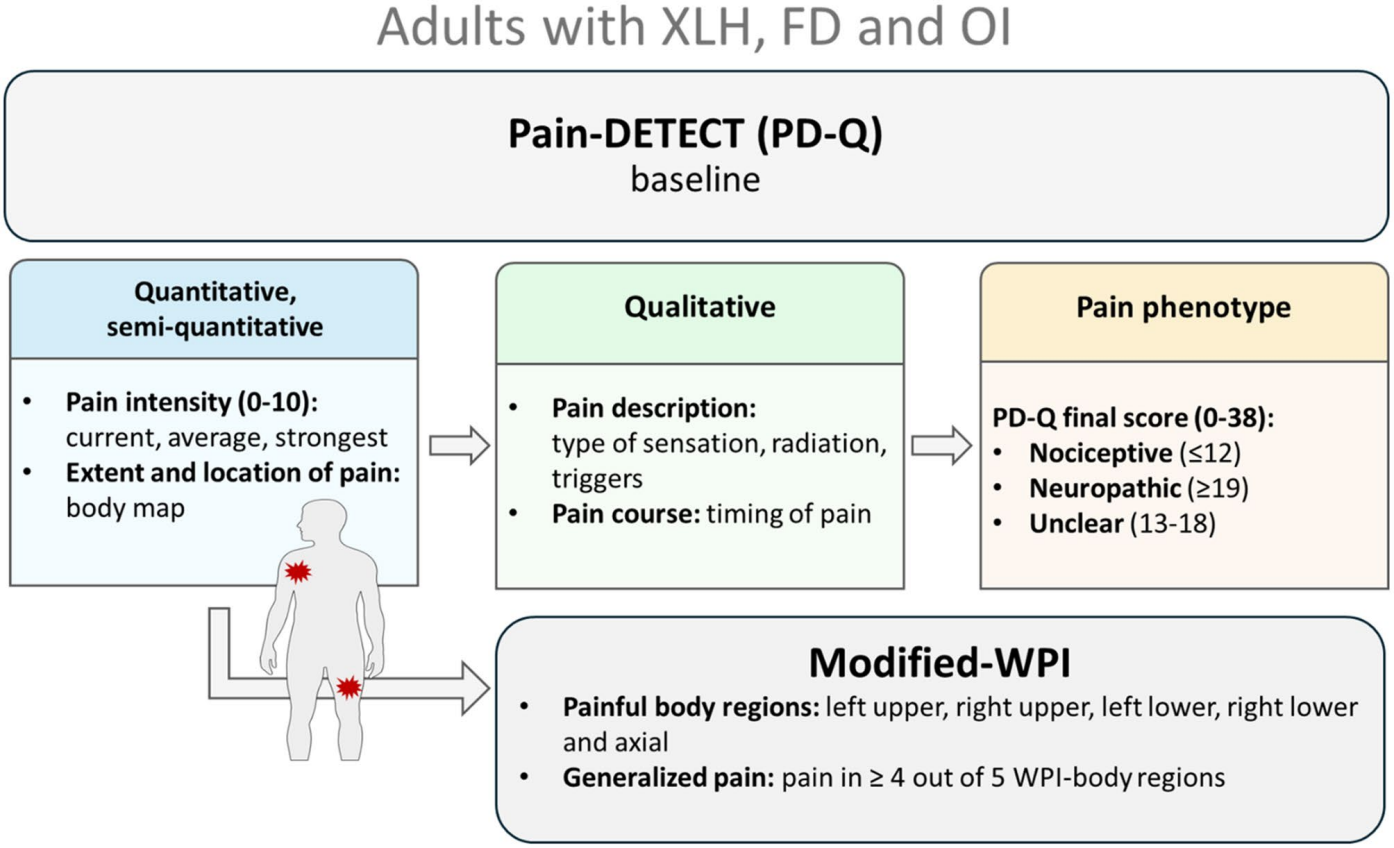
Pain assessement tools. Pain assessment in this study was performed multimodally using the PainDETECT Questionnaire (PD-Q) at baseline and a modified Widespread Pain Index (WPI) in adults with XLH, FD, and OI. The PD-Q provided quantitative measures, including current, average, and strongest pain intensity over the past 4 weeks, as well as semi-quantitative measures of pain extent and number of painful locations using a body map. It also allowed qualitative assessment, with the final score defining pain phenotype (0–12: nociceptive; 13–18: unclear; 19–38: neuropathic), pain description (type of sensation such as tingling, burning, or electric shocks; triggers; radiation), and pain course (e.g., persistent pain with attacks). The complete PD-Q version from RUDY.test.org is provided in Supplemental Fig. 1. The modified WPI, derived from the PD-Q body map, was used to evaluate generalized pain and the nociplastic component. Additional details on the modified WPI are available in Supplemental Table 1

The PD-Q (Fig. 1, supplemental Fig. 1) is a 12-item questionnaire designed originally designed as a screening tool for neuropathic pain in the context of back pain [6]. It was later adapted for use in knee osteoarthritis [7] and has also been applied in numerous other musculoskeletal conditions [8], including FD/MAS [9], OI [10] or XLH [11, 12]. There is increasing evidence that it may also capture centrally mediated pain mechanisms, including nociplastic pain. Pain phenotype was determined based on the final PD-Q score as follows: nociceptive (score 0–12), “unclear” (score 13–18), or neuropathic-like (score 19–38), as previously described [6, 9]. In addition to pain characterization, the PD-Q has three separate questions which allow for the quantification of pain intensity on a scale from 0 to 10, recording current pain, average pain and the strongest pain experienced in the past four weeks. Current pain prevalence was defined as the proportion of patients reporting pain within the previous 24 h, while severe strongest pain prevalence corresponded to the proportion of patients describing their strongest pain intensity as greater than or equal 8/10 within the past month. Patients were also able to report the course of their pain by selecting one of the following profiles: persistent pain with slight fluctuations, persistent pain with pain attacks, pain attacks without pain between them or, pain attacks with pain between pain them. The online PD-Q version (supplemental Fig. 1) includes a human body map that enables patients to indicate pain locations and if the pain radiates. For the purpose of the study, we defined four clinically relevant regions: craniofacial (face, jaw, forehead and headback), lower limbs (hip, buttock, thigh, knee, calf, ankle, foot), upper limbs (shoulder, upper arm, elbow, forearm, wrist and hand), and axial (chest, cervico-thoraco-lumbar spine and abdomen). Patients were able to report pain in any or all of the four areas.

The Widespread Pain Index (WPI) is used to assess widespread pain forms part of the 2016 revised ACR fibromyalgia diagnostic criteria [13], alongside the symptom severity scale (SSS) score. The WPI assesses the presence of pain in 19 specific body locations, including jaw, upper and lower limbs, as well as the axial areas. The self-report version of the fibromyalgia diagnostic criteria has been validated for epidemiologic studies [13]. According to these criteria, generalized pain is defined as pain in at least 4 out of 5 WPI body regions (left upper region, right upper region, left lower region, right lower region and axial region), excluding pain in the chest, jaw and the abdomen. Pain location data from the PD-Q body map were used to create an equivalent WPI (Fig. 1, supplemental Table 1) and allowing estimation of the prevalence of generalized pain. The axial region was slightly modified in this equivalent WPI because the back areas were labeled and divided differently (lower/upper back in WPI vs. back and spine in modified WPI), although the entire back/spine region was similarly covered.

Anxiety and depression were evaluated using baseline Hospital Anxiety and Depression Scale (HADS) [14, 15] and defined based on following score in the last week separately for anxiety and depression: scores below 8 was considered normal, 8 to 10 indicate mild anxiety or depression, 11 to 14 moderate and scores from 15 to 21 reflected severe anxiety or depression. Sleep was assessed by baseline Pittsburgh Sleep Quality Index (PSQI) in the past 4 weeks [16, 17], with sleep impairment defined by a score greater than 8 [9].

### Statistical analysis

Descriptive statistical analyses were conducted using R^®^ and GraphPad Software^®^ to summarize patient characteristics and pain, mental health, and sleep outcomes. Unadjusted comparisons of outcomes across the three rare bone diseases (FD, OI, and XLH) and between pain phenotypes (nociceptive, neuropathic-like, unclear) were performed using the Kruskal-Wallis test for continuous variables and the Chi-squared test for categorical variables. Continuous variables are reported as median (interquartile range) and categorical variables as n (%). When statistically significant (*p* < 0.05), post-hoc pairwise comparisons were performed using Dunn’s test for continuous variables and Fisher’s exact test for categorical variables, with Bonferroni correction for multiple testing. The Wilcoxon rank-sum test was used for subgroup analyses.

Multivariate analyses were performed to evaluate the associations of pain phenotype with pain, mental health, and sleep outcomes, adjusting for age and sex. Continuous outcomes were analyzed using linear regression (reported as regression coefficients with 95% confidence intervals [CI]) and binary outcomes using binomial regression (reported as odds ratios [ORs] with 95% CI), with nociceptive pain as the reference category. Independent effects of age and sex were also assessed in separate models using linear, binomial regression and multinomial regression, according to the outcome. Females were used as the reference group.

## Results

### Patients characteristics

We included a total of 281 adults who completed the PD-Q at baseline: 94 patients with FD/MAS, 94 with OI, and 93 with XLH. Regarding demographic characteristics, the median age was 44 years (range ,18–78), and 73% of the cohort were women, with no differences in age or sex between the three diseases (Table 1).

**Table 1:** Demographics and pain characteristics in adult patients with FD/MAS, OI, and XLH

	Overall (*n* = 281)	FD/MAS (*n* = 94)	OI (*n* = 94)	XLH (*n* = 93)	*p*
Demographic factors					
Median age, years	44(18–78)	47(18–74)	43(21–75)	41(19–78)	0.17
Women	205(73%)	67(71%)	69(73%)	69(74%)	0.9
**Pain intensity**					
Current pain	4(1–6)	4.5(1.25-7)	3(1–5)	4(2–7)	0.09
Average pain in last 4 weeks	5(3–7)	5(3–7)	4(3–6)	5(3–7)	0.07
Strongest pain in last 4 weeks	7(5–9)	8(5–9)†	6(4–8)	8(5–9)	0.03*
**Pain occurrence (y/n)**					
Current pain	243(86%)	81(86%)	82(87%)	80(86%)	0.97
Strongest pain ≥ 8/10 in last 4 weeks	132(47%)	50(53%)	35(37%)	47(51%)	0.06
**Pain phenotype**					0.63
- nociceptive	172(64%)	54(60%)	63(70%)	55(62%)	
- neuropathic	38(14%)	15(17%)	11(12%)	12(13%)	
- unclear	59(22%)	21(23%)	16(18%)	22(25%)	
- missing data	12(4%)	4(4%)	4(4%)	4(4%)	
**Pain location**					
- cranio-facial	41(15%)	26(28%)†°	8(9%)	7(8%)	< 0.01*
- upper-limb	126(45%)	34(36%)	49(52%)	43(46%)	0.08
- lower-limb	230(82%)	60(64%)†°	83(88%)	87(94%)	< 0.01*
- axial	161(57%)	39(42%)	71(76%)†‡	51(55%)	< 0.01*
**Extent of painful location**					
Number of painful areas (1–66)	6(2–12)	3(2-5.75)**†°**	7(4–14)	9(4-15.5)	< 0.01*
Modified-WPI generalized pain (y/n)	51(18%)	11(12%)	20(21%)	20(22%)	0.14
**Time course of pain**					0.1
Persistent pain with slight fluctuations	61(23%)	15(17%)	26(29%)	20(22%)	
Persistent pain with pain attacks	85(32%)	36(40%)	19(21%)	30(34%)	
Pain attacks without pain between them	74(28%)	26(29%)	28(31%)	20(22%)	
Pain attacks with pain between them	49(18%)	13(14%)	17(19%)	19(21%)	
Missing datas	12(4%)	4(4%)	4(4%)	4(4%)	
**Anxiety and depression**,** HADS**					
Anxiety score	7(4–11)	6(3-10.25)	6(3-9.75)	9(6–12)‡°	< 0.01*
Moderate or severe anxiety	55(27%)	17(25%)	13(21%)	25(35%)	0.18
Depression score	5(2–9)	4(1-9.25)	4(2–7)	7(4–10)‡°	0.01*
Moderate or severe depression	38(19%)	14(21%)	7(11%)	17(24%)	0.19
Missing data	80(28%)	26(28%)	32(34%)	22(24%)	
**Quality of sleep**,** PSQI**					
Score	9(6–12)	10(6–13)	8(1–11)	9(6–12)	0.46
Sleep impairment	144(55%)	50(56%)	45(51%)	49(57%)	0.84
Missing datas	17(6%)	5(5%)	5(5%)	7(8%)	

Comparison of demographics, pain, mental health, and sleep outcomes in patients across the three rare bone diseases FD, OI, and XLH. Kruskal-Wallis or Chi-squared tests were used, with Dunn’s or Fisher’s exact for post-hoc analyses as appropriate. Data are shown as median (IQR) or n (%),unless otherwise indicated. y/n : yes or no

* *p* < 0.05, comparison between the 3 diseases

† *p* < 0.05, comparison FD/MAS vs. OI

˚*p* < 0.05, comparison FD/MAS vs. XLH

‡ *p* < 0.05, comparison OI vs. XLH

Abbreviations: FD/MAS: Fibrous Dysplasia/McCune-Albright; HADS: Hospital Anxiety and Depression Scale ; med = median ; IQR : Interquartile Range ; OI: Osteogenesis Imperfecta; PSQI : Pittsburgh Sleep Quality Index ; RUDY: Rare and Undiagnosed Diseases study; XLH: X-linked Hypophosphatemia; WPI: Widespread Pain Index

### Pain distribution and characteristics

#### Pain frequency

86% of patients from the overall cohort reported experiencing current pain, with similar proportions across the three diseases (*p* = 0.97): 86% of FD patients, 87% of OI patients and 86% of XLH patients. 47% of patients reported their strongest pain in the past month as severe (≥ 8/10), with no statistically significant differences with patients in the OI group (37%) than FD/MAS (53%) and XLH (51%) groups (Table 1). 18% of the overall cohort had generalized pain as determined by the modified-WPI, with no significant difference between the disease groups (*p* = 0.14).

#### Pain intensity

**Fig. 2:**
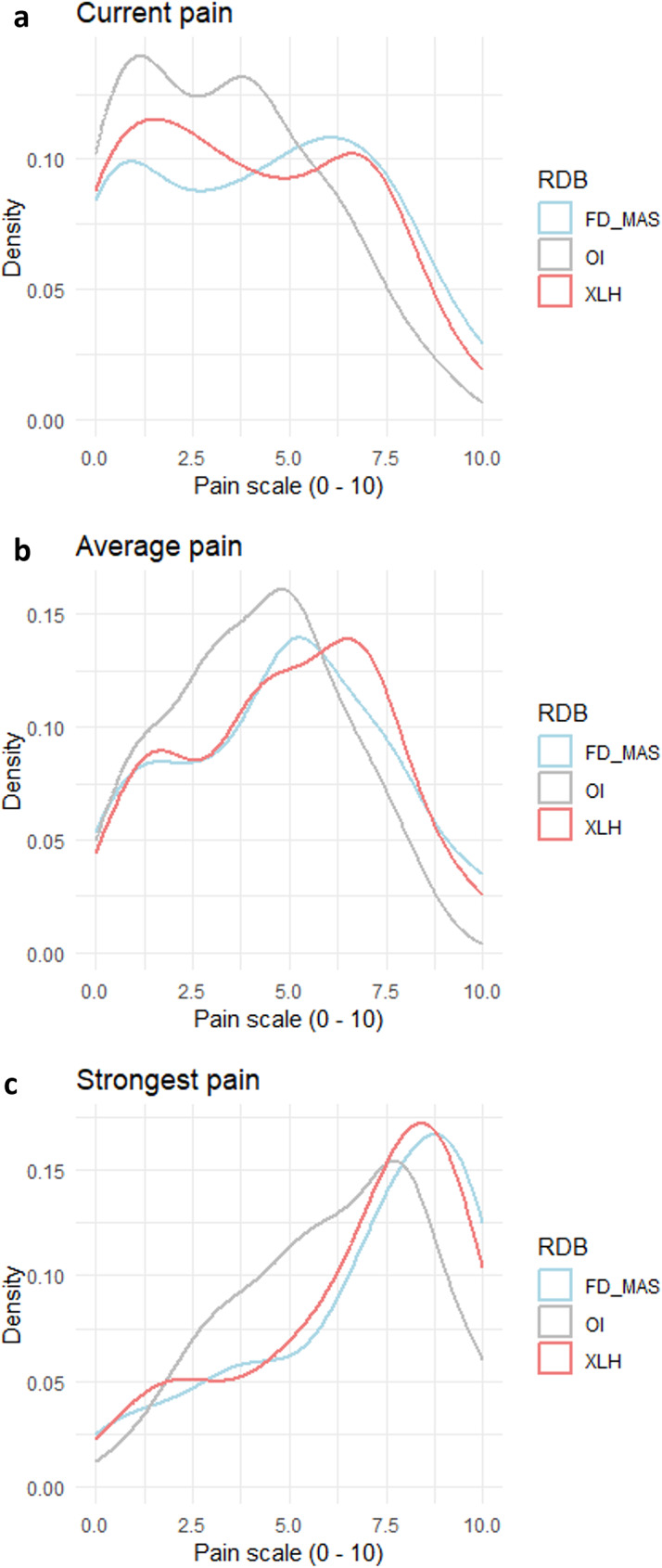
Distribution of pain intensity in FD/MAS, OI and XLH

There was no significant difference in current or average pain intensity (Fig. 2) between disease groups (*p* = 0.09). However, the intensity of the strongest pain experienced in the past four weeks differed significantly between the three diseases (*p* = 0.03) and was significantly lower (median (IQR): 6 (4-8)) in OI patients compared to those with FD/MAS (8 (5-9), *p* = 0.02).

#### Pain phenotype

**Fig. 3:**
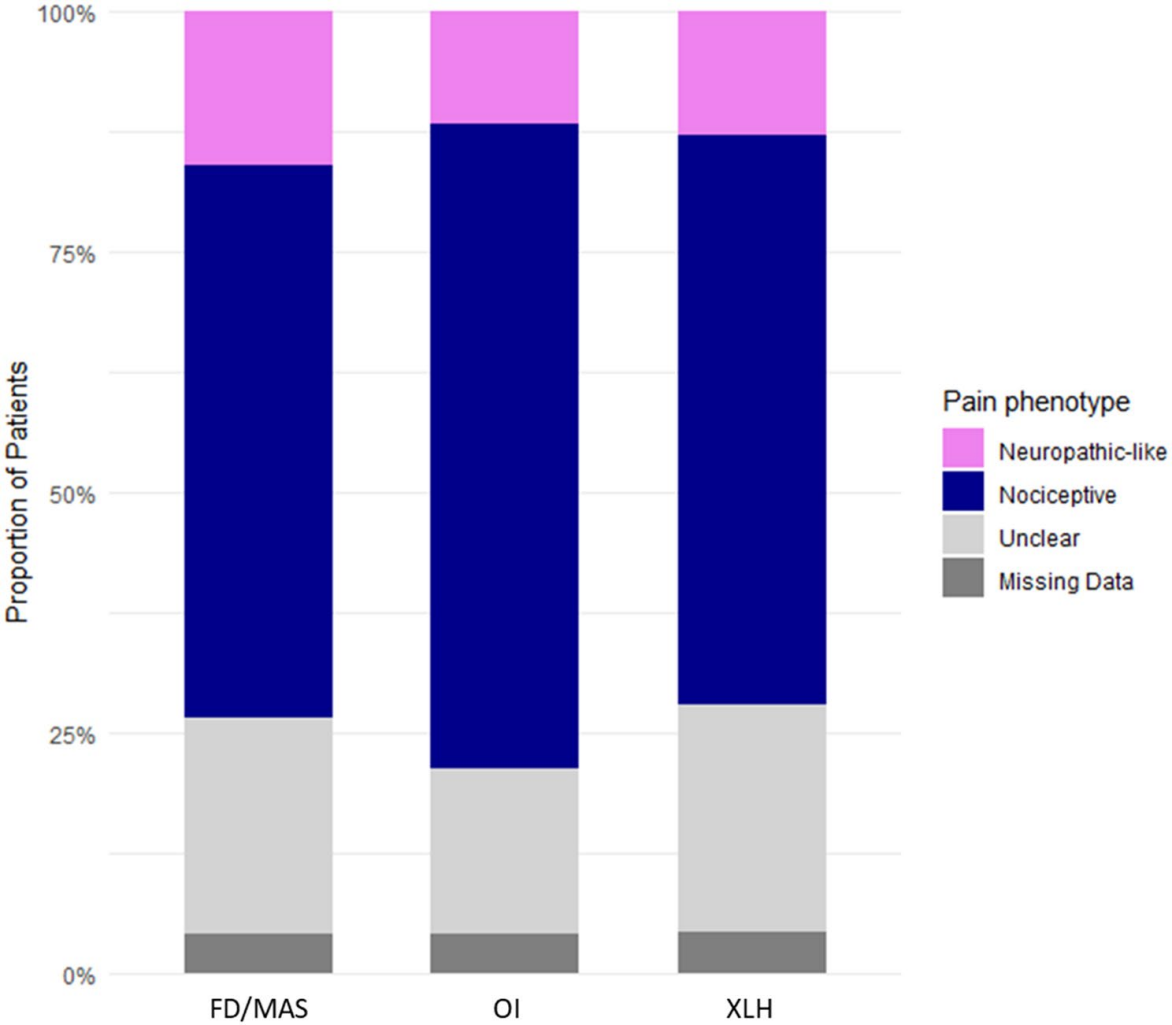
Proportions of nociceptive, unclear, and neuropathic-like pain profiles across each rare bone disease. The pain profile—categorized as nociceptive, unclear, or neuropathic-like pain was determined on the baseline PD-

Among pain phenotypes, nociceptive pain was the most common (64%), followed by “unclear” pain (22%) and neuropathic-like pain (14%), with no differences in proportions between the 3 types of disease (*p* = 0.6, Table 1; Fig. 3). Adults reporting neuropathic-like or “unclear” phenotype had significantly higher pain levels (*p* < 0.001) and more frequently reported severe strongest pain (*p* < 0.001) compared to those with a nociceptive profile (Table 2a and 2b).

**Table 2a:** Pain outcomes and quality of life measures, according to pain phenotype

	nociceptive (*n* = 172)	neuropathic-like (*n* = 38)	unclear (*n* = 59)	*p*
**Rare bone disease**				0.63
FD/MAS	54(31%)	15(39%)	21(36%)	
OI	63(37%)	11(29%)	16(27%)	
XLH	55(32%)	12(32%)	22(37%)	
**Pain intensity (0–10)**				
Current pain	2(1–4)^a, b^	7(5–8)	6(4–7)	< 0.01*
Average pain	4(2–5)^a, b^	7(6–8)	6(5–7)	< 0.01*
Strongest pain	6(4–8)^a, b^	9(8–10)	9(8–9)	< 0.01*
**Pain occurence (y/n)**				
Current pain (y)	144(84%)^b^	37(97%)	58(98%)	< 0.01*
Severe strongest pain (y)	50(29%)^a, b^	34(39%)	47(80%)	< 0.01*
**Extent of painful location**				
Number of painful areas (1–66)	4(2–8)^a, b^	15.5(8.5–25)	10(4.25-16)	< 0.01*
Modified-WPI-generalized pain	16(9%)^a, b^	17(45%)	17(29%)	< 0.01*
**Anxiety and depression**,** HADS**				
Anxiety score (0–21)	6(3–10)	12(10–15)^a, c^	8(5–11)	< 0.01*
Moderate/severe anxiety (y)	24(19%)	17(63%)^a, c^	12(29%)	< 0.01*
Depression score (0–21)	4(2–7)^a, b^	11(7.5–13.5)	8(5–12)	< 0.01*
Moderate/severe depression (y)	10(8%)^a, b^	14(52%)	13(32%)	< 0.01*
Missing datas	48(28%)	11(29%)	18(31%)	
**Quality of sleep**,** PSQI**				
Score (0–21)	8(5–10)^a, b^	14(10–17)^c^	11.5(8–14)	< 0.01*
Sleep impairment (y)	74(45%)^a, b^	30(86%)	38(70%)	< 0.01*
Missing datas	8(5%)	3(8%)	5(8%)	

Comparison of rare bone disease type, pain outcomes, mental health, and sleep parameters according to pain phenotype (nociceptive, neuropathic-like, unclear). Continuous variables are reported as median (interquartile range) and compared using the Kruskal–Wallis test with Dunn’s post-hoc analysis. Categorical variables are presented as n (%) and compared using Chi-square or Fisher’s exact test, as appropriate. y/n : yes or no ; y : yes

* *p* < 0.05, comparison between the 3 phenotypes (nociceptive, neuropathic-like and unclear)

a *p* < 0.05, comparison neuropathic-like vs. nociceptive

b *p* < 0.05,comparison unclear vs. nociceptive

c *p* < 0.05, comparison neuropathic-like vs. unclear

**Table 2b:** Association of pain phenotype with quality of life and pain outcomes: multivariate model adjusted for age and sex

	Neuropathic-like		Unclear	
Pain intensity	Coefficient	95% CI	Coefficient	95% CI
Current pain	3.63	2.81 ; 4,45	2.53	1,84 ; 3,22
Average pain	3.30	2.56 ; 4.03	2.24	1.62 ; 2.85
Strongest pain	2.91	2.15 ; 3.66	2.59	1.95 ; 3.23
**Anxiety and depression (y/n)**	**OR**	**95% CI**	**OR**	**95% CI**
Moderate/severe anxiety	6.75	2.74 ; 17.48	1.64	0.70 ; 3.72
Moderate/severe depression	12.08	4.51 ; 34.23	5.47	2.15 ; 14.41
**Sleep impairment (y/n)**	**OR**	**95% CI**	**OR**	**95% CI**
	7.23	2.89 ; 22.06	2.85	1.49 ; 5.66

Separate multivariate models, each adjusted for age and sex, were used to evaluate the predictive value of pain phenotype, with nociceptive pain as the reference category. Binary outcomes (moderate/severe anxiety or depression, and sleep impairment) were analyzed using binomial regression and are reported as odds ratios (ORs) with 95% confidence intervals (95% CI). Continuous outcomes (pain intensity) were analyzed using linear regression and are reported as regression coefficients with 95% CI. y/n : yes or no

#### Evolving pain profile

The commonest pain course was “persistent pain with pain attacks” (32%), following “pain attacks without pain between them” (28%), with no significant difference between the 3 diseases (*p* = 0.1, Table 1).

#### Painful sites

**Fig. 4:**
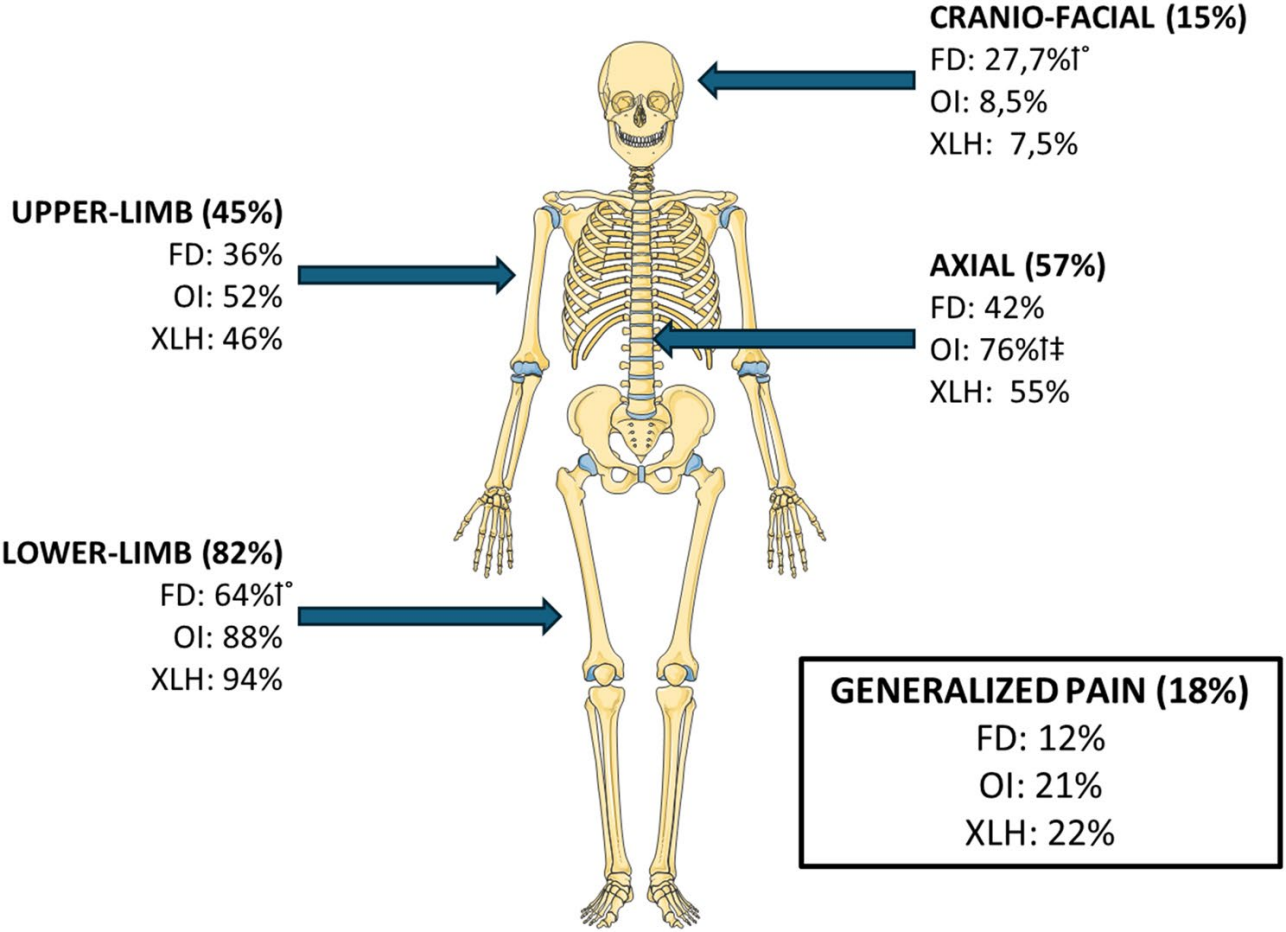
Pain location. Proportion of patients reporting pain in the upper limbs, lower limbs, axial skeleton, craniofacial region, or generalized pain, shown on a body map based on the PD-Q. Results are presented for all patients and by diagnostic group (XLH, FD, and OI). † *p* < 0,05, comparison FD/MAS vs. OI. ° *p* < 0,05, comparison FD/MAS vs. XLH. ‡ *p* < 0,05, comparison OI vs. XLH

Overall, the lower limbs were the most frequently reported pain site among patients (82%), followed by the axial skeleton (57%) (Fig. 4). The sites of body pain differed between the rare bone diseases. In FD, a significantly lower proportion of patients reported pain in the lower limbs (64%), compared to adults with OI (88%, *p* < 0.001) and XLH (94%, *p* < 0.0001). Conversely, a significantly higher proportion of FD patients reported pain at the craniofacial site (28%), in comparison to adults with OI (9%, *p* < 0.01) or XLH (8%, *p* < 0.01). Axial pain was significantly more frequent in patients with OI (76%), compared to FD patients (41%, *p* < 0.0001) or XLH patients (55%, *p* < 0.05). The number of painful areas reported on the PD-Q body map (1–66) was significantly lower in FD patients (median(IQR): 3 (2-5.75)) compared to patients with OI (median(IQR): 7 (4-14), *p* < 0.001) or XLH (median(IQR): 9 (4-15.5), *p* < 0.001) (Table 1).

#### Influence of age and sex

To determine the independent effects of age or sex on each pain outcome, each was separately assessed using distinct multivariate models in the overall cohort of 281 patients (Table 3). Age showed no association with any pain outcome, including pain occurrence, pain level, number of painful sites, or phenotype. In contrast, sex was associated with multiple pain outcomes, including both pain occurrence and pain levels. Males were less likely to report having pain, with a lower likelihood of both current pain (OR [95% CI]: 0.40 [0.20–0.82], *p* = 0.01) and severe pain in the past 4 weeks (OR [95% CI]: 0.42 [0.24–0.73], *p* < 0.01), compared to females. Male sex was also consistently associated with lower pain intensities than female sex for current pain (Coefficient[CI95%]: -0.89, 95% CI: -1.62 to -0.16), average (-0.94, 95% CI: -1.60 to -0.28), and strongest pain (-1.31, 95% CI: -2.01 to -0.61) reported within the past 4 weeks (all *p* < 0.05). However, there were no sex differences in the extent of painful sites or pain phenotype.

In addition, associations between age or sex and pain outcomes were consistent across the different rare bone diseases, as no significant interactions were found in the models, nor in analyses stratified by age (data not shown).

**Table 3:** Independent effects of age and sex on pain outcomes in overall cohort

	Age			Male sex		
**Pain occurrence (y/*n***)	**OR**	**95% CI**	***p***	**OR**	**95% CI**	***p***
Current pain (y)	0.99	0.97 ; 1.02	0.53	0.40	0.20 ; 0.82	0.01*
Severe strongest pain in past 4 weeks (y)	0.99	0.98 ; 1.01	0.41	0.42	0.24 ; 0.73	< 0.01*
Generalized pain (y)	1.00	0.98 ; 1.02	0.93	0.60	0.27 ; 1.24	0.19
**Pain intensity**	**Coefficients**	**95% CI**	**p**	**Coefficients**	**95% CI**	**p**
Current pain	-0.014	-0.04 ; 0.01	0.21	-0.89	-1.62 ; -0.16	0.02*
Average pain in past 4 weeks	-0.013	-0.03 ; 0.01	0.18	-0.94	-1.60 ; -0.28	< 0.01*
Strongest pain in past 4 weeks	-0.014	-0.03 ; 0.01	0.20	-1.31	-2.01 ; -0.61	< 0.01*
**Extent of painful location**	**Coefficients**	**95% CI**	**p**	**Coefficients**	**95% CI**	**p**
Number of painful areas (1–66)	-0.033	-0.10 ; 0.03	0.28	-1.36	-3.60 ; 0.87	0.23
**Pain phenotype**	**OR**	**95% CI**		**OR**	**95% CI**	
Neuropathic	1.00	0.97 ; 1.02		0.68	0.29 ; 1.59	
Unclear	1.01	0.99 ; 1.03		0.57	0.27 ; 1.18	

The independent effects of age and sex on each pain outcome were evaluated using distinct models in the overall cohort of 281 patients, with female sex and nociceptive pain as reference categories. Pain occurrence (yes/no) was analyzed using binomial regression (OR, 95% CI), continuous outcomes (pain intensity, number of painful sites) using linear regression (coefficients, 95% CI), and pain phenotype using multinomial regression (OR, 95% CI). Males were less likely to report having pain, both current (OR = 0.40 [95% CI 0.20–0.82], *p* = 0.01) and severe in the past 4 weeks (OR = 0.42 [95% CI 0.24–0.73], *p* < 0.01), and reported lower pain intensities across all measures (all *p* < 0.05), compared to females. However, there were no sex differences in the extent of painful sites or pain phenotype, and age was not associated with any pain outcome. y/n : yes or no ; y : yes. *p < 0.05

#### Pain characteristics by disease

Sex was not associated with any pain outcomes in the FD/MAS group, and age was not linked to any pain parameters. Monostotic/polyostotic status was reported for 59 (63%) of the patients. Pain levels, pain frequency, and pain phenotype, as well as the pain course profile, were similar between monostotic and polyostotic FD (supplemental Table 2). 2 out of 27 monostotic patients reported generalized pain according to the modified WPI, despite being expected to experience pain only at their single bone site lesion.

Contrary to FD, sex was associated with different pain outcomes in OI, with females being more likely to experience severe pain (Supplemental Table 3). Age was not associated with any pain parameters. The relationship between sex and pain phenotype could not be assessed (supplemental table 3b) due to the absence of males with neuropathic pain in this limited sample. Although the overall distribution of pain course profiles differed significantly between females and males (*p* = 0.03, supplemental table 3a), no significant differences were found when each profile was examined separately.

In XLH adults (supplemental Table 4), female sex was only associated with current pain prevalence. Age was not associated to any pain parameters.

### Quality of life measurements

In the overall cohort, 27% of patients reported moderate to severe anxiety and 19% moderate to severe depression, with no significant difference between the three diseases (*p* = 0.19) (Table 1). XLH patients exhibited poorer mental well-being outcomes, with a significantly higher median HADS score for anxiety compared to patients with FD/MAS (*p* < 0.01) or OI (*p* < 0.01). XLH patients also had a significantly higher median HADS score for depression than patients with FD/MAS (*p* = 0.01) or OI (*p* = 0.01). Regarding sleep quality, 55% of the cohort reported sleep impairment on the PSQI, with similar proportion between the 3 conditions (*p* = 0.84). No significant difference in the median PSQI score was observed between the three diseases (*p* = 0.84).

In multivariate models, a neuropathic-like profile was associated with both moderate to severe anxiety and depression, as well as sleep impairment (Table 2b). “Unclear” pain profile was associated with moderate to severe depression and sleep impairment but not with moderate to severe anxiety.

As described above, 18% of the overall cohort had generalized pain according to the modified WPI. Additional analyses of the full cohort of patients showed that generalized pain was associated with significantly higher pain levels and poorer quality of life parameters compared to patients without generalized pain (Table 4). Pain levels (current pain, strongest and average pain within 4 weeks) were significantly higher in patients experiencing generalized pain in comparison to those without generalized pain (*p* < 0.0001). Patients with generalized pain were more likely to experience neuropathic-like pain or “unclear” pain than nociceptive pain (respectively, *p* < 0.0001 and *p* < 0.01) (Fig. 5a). Anxiety and depression score were significantly higher in patients with generalized pain than patients without generalized pain (respectively *p* < 0.001 and *p* < 0.0001). The proportion of patients having moderate to severe anxiety was also significantly higher in case of generalized pain (43% vs. 24%, *p* = 0.03) as the proportion of moderate to severe depression (41% vs. 14%, *p* < 0.001) – Figs. 5b and 5c. Regarding sleep, median score of PSQI was significantly higher in patients with generalized pain (*p* < 0.0001) and the proportion of patients with sleep impairment were significantly higher in generalized pain (78% vs. 50%, *p* < 0.001) (Fig. 5d).

**Table 4:** Pain characteristics and quality of life outcomes among patients with generalized pain

	No Generalized pain (*n* = 230)	Generalized pain (*n* = 51)	*p*
Demographic characteristics			
Women	164(71%)	41(80%)	0.25
Age, years (range)	44(18–78)	43(22–78)	0.99
**Rare bone disease**			0.14
FD/MAS	83(36%)	11(22%)	
OI	74(32%)	20(39%)	
XLH	73(32%)	20(39%)	
**Pain-detect intensity**			
Current pain intensity	3(1–6)	6(4–7)	< 0.01*
Strongest pain intensity	7(4–8)	8(7–9)	< 0.01*
Average pain intensity	4.5(2–6)	6(4.5-7)	< 0.01*
**Pain phenotype**			< 0.01*
Neuropathic-like	21(10%)^a^	17(34%)	
Nociceptive	156(71%)^a, b^	16(32%)	
Unclear	42(19%)	17(34%)	
Missing datas	11(5%)	1(2%)	
**HADS (** ***n*** ** = 201)**			
Anxiety score (0–21)	6(3–10)	10(7–13)	< 0.01*
Severe/moderate anxiety (y)	39(24%)	16(43%)	0.03*
Depression score (0–21)	4(2–9)	9(6–12)	< 0.01*
Severe/moderate depression (y)	23(14%)	15(41%)	< 0.01*
Missing datas	66(29%)	13(25%)	
**Sleep (** ***n*** ** = 264)**			
PSQI score (0–21)	8(5.3–12)	11(8.3–14)	< 0.01*
Sleep impairment (y)	108(50%)	36(78%)	< 0.01*
Missing datas	12(5%)	5(10%)	

Comparison of pain characteristics and quality-of-life outcomes in patients with and without generalized pain, using the Wilcoxon rank-sum test and Chi-square or Fisher’s exact test, as appropriate. Data are expressed as median (IQR) or n (%), unless otherwise indicated. y : yes

* *p* < 0.05, comparison between patients with generalized pain versus those without

a *p* < 0.05, comparison neuropathic-like vs. nociceptive

b *p* < 0.05,comparison unclear vs. nociceptive

**Fig. 5a:**
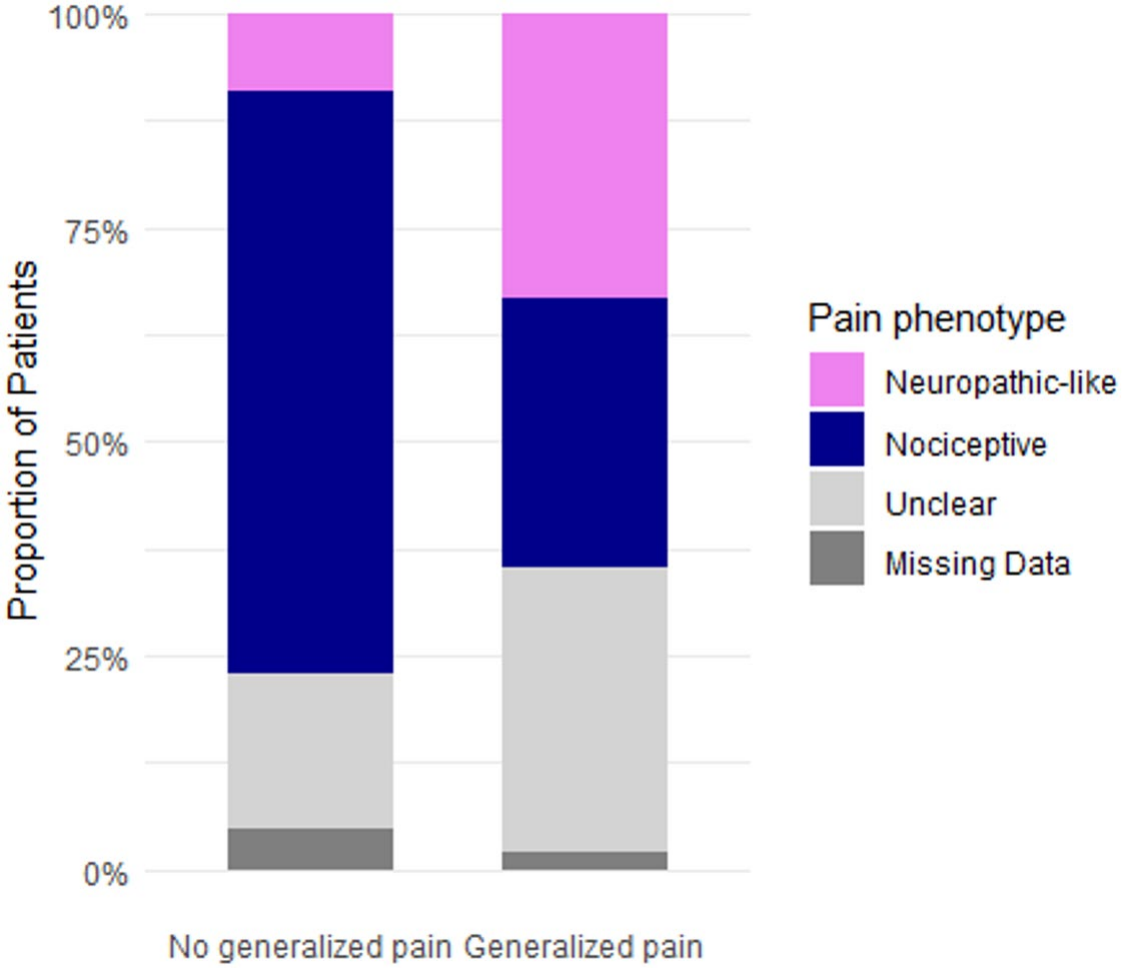
Impact of Generalized pain on pain phenotype and quality of life outcomes in adults with rare bone diseases. Proportions of nociceptive, unclear, and neuropathic-like pain profiles according to generalized pain status Proportion of individuals in each pain profile (nociceptive, unclear, or neuropathic-like), as defined by the baseline PD-Q, among patients with or without generalized pain according to the modified-WPI

**Fig. 5b:**
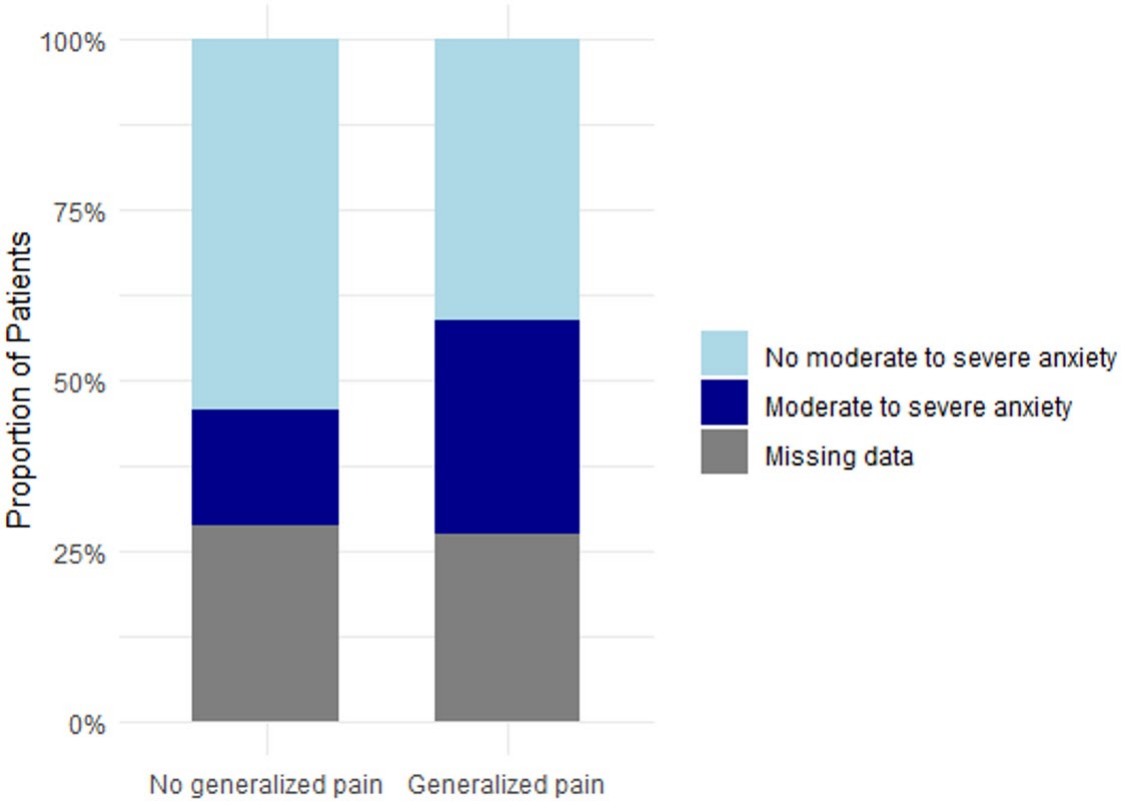
Proportions of moderate to severe anxiety based on generalized pain status Proportion of individuals with moderate to severe anxiety, as assessed by the HADS, among patients with or without generalized pain according to the modified-WPI

**Fig. 5c:**
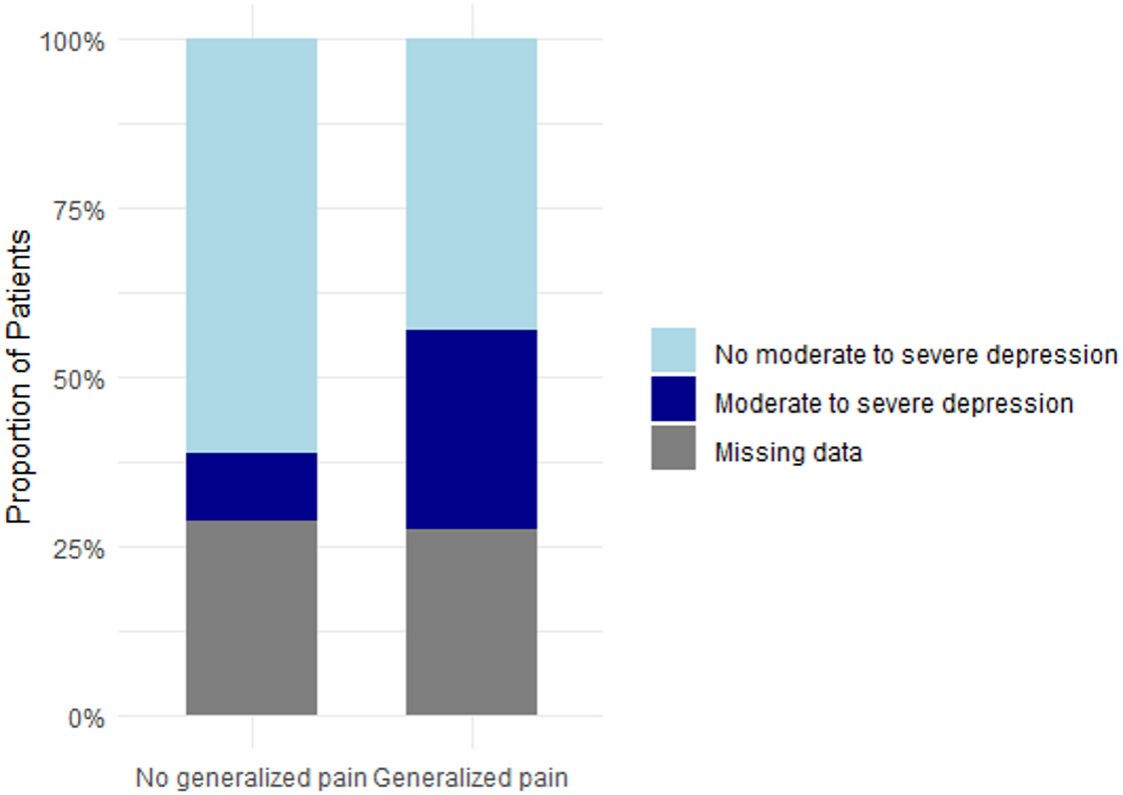
Proportions of moderate to severe depression based on generalized pain status Proportion of individuals with moderate to severe depression, as assessed by the HADS, among patients with or without generalized pain according to the modified-WPI

**Fig. 5d:**
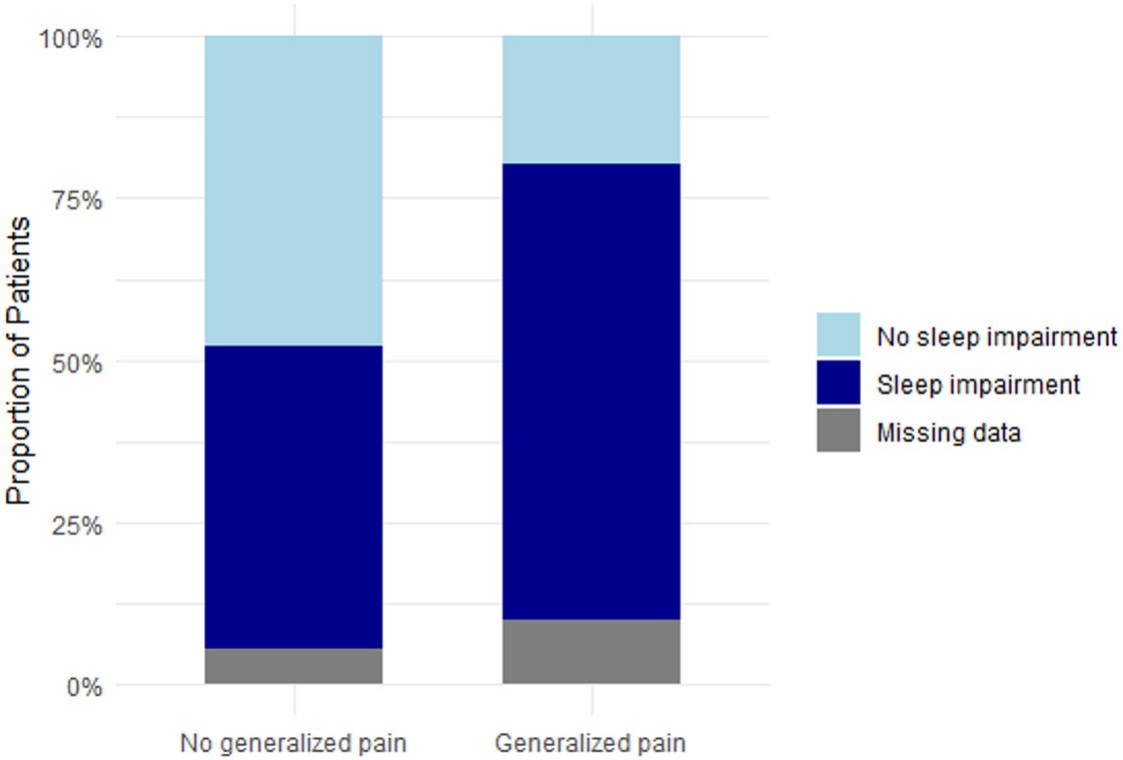
Proportions of sleep impairment based on generalized pain status Proportion of individuals with sleep impairment, as assessed by the PSQI, among patients with or without generalized pain according to the modified-WPI

## Discussion

This cross-sectional study compared pain characteristics in three rare bone diseases – FD/MAS, OI and XLH - and confirmed the unmet challenge of pain management in rare diseases as evidenced by the high prevalence of pain across conditions. In this study, current pain was experienced by 86% of patients and 47% of patients describing their strongest pain within the past month as severe.

Given the distinct pathophysiological mechanisms of these three rare bone diseases, we anticipated identifying differences and specific features in pain parameters for each condition. However, most pain outcomes (pain prevalence, intensity, phenotype and time course) were remarkably stable across the three diseases, suggesting that extra-osseous factors are the likely primary drivers for these aspects of pain. Conversely, pain sites appeared to be disease-specific. Previous study has shown that the anatomical location of pain may influence its frequency as well as its severity [18]. In our study, while age did not appear to influence pain, sex was an important determinant, with women being more likely to experience pain and severe pain, as previously described in chronic pain [19–21]. Evidence of sex differences in pain perception has been reported, involving distinct genes, proteins, and hormonal-immune system interactions, as well as differences in CNS pain pathways, coping strategies, and psychosocial factors, which modulate pain differently in males and females. In rare bone diseases, higher prevalence of pain have been reported in females in FD/MAS [18] and in OI [10, 22] but sex was not correlated with pain severity. Although age-related changes in pain perception and modulation have been suggested in patients with chronic pain or in healthy subjects [23–25], we did not find an association between pain outcomes and age in the specific population of rare bone diseases. In this population, most studies have reported that adults are more likely to experience pain and higher pain levels than children [26–31], probably due to the cumulative burden of painful events over the lifespan (e.g., fractures, surgeries, osteoarthritis). However, findings among adults with rare bone disease are inconsistent: while some studies describe a worsening of pain with increasing age [10, 22], others report no significant age-related differences in pain outcomes during adulthood [9, 18, 32, 33], perhaps because cohorts of rare disease patients include few older individuals.

In this study, the most prevalent pain phenotype was nociceptive pain, present in up to 64% of the overall cohort, with no significant difference among the 3 diseases. The pain phenotype showed a consistent and independent association, with neuropathic-like and “unclear” profiles associated with greater pain severity and poorer quality-of-life measurements, including anxiety, depression, and sleep impairment. Previous studies have described the prevalence of neuropathic-like pain in patients with FD/MAS [9], in OI [10] and in XLH [11, 12]. Among these, only one study, conducted in FD/MAS patients has identified an association between neuropathic-like pain and worse pain severity, higher levels of anxiety and depression, impaired quality of life, and sleep disturbances—findings that we confirmed in our study. Neuroimaging findings also support the association between neuropathic pain and pain severity in FD/MAS [34]. In our study, the “unclear” phenotype, which represents 22% of the overall cohort, may result from a mixed pattern of neuropathic-like and nociceptive pain types. Although this has not been proven, we cannot rule out that the “unclear” profile may be related to a nociplastic pattern as well as a proportion of patients with neuropathic-like pain.

Nociplastic pain is a common complication of chronic pain in rheumatic conditions, with the prevalence of fibromyalgia ranging from 10% to 48% in patients with rheumatic diseases [35, 36]. Nociplastic pain is characterized by widespread body pain, often concomitantly associated with fatigue and altered sleep, cognition and mood, as well as multisensory hypersensitivity [37]. Recently recognized as the third category of pain, nociplastic pain can coexist with nociceptive and/or neuropathic pain, potentially forming a continuum [37]. The PD-Q, initially developed to diagnosed neuropathic pain can also be applied for nociplastic pain [38]. Neuropathic-like pain detected by the PD-Q has been correlated with central sensitization [39, 40], which is also a key mechanism driving nociplastic pain. Neuropathic pain may be more reflective of nociplastic mechanisms, given the lack of evidence for peripheral nerve damage or sensitization [41], in contrast with emerging evidence for central nervous system changes [34] in rare bone diseases. Moreover, the consistency of pain characteristics and distribution across diseases with distinct skeletal pathologies and manifestations prompts consideration of on-skeletal mechanisms—such as central sensitization or nociplastic pain—may play a predominant role in the pain experience in this population. This hypothesis of nociplastic mechanisms is further supported by the high prevalence of generalized pain (18%) in our cohort, as assessed using the modified-WPI, along with association with moderate to severe anxiety and depression, as well as sleep disturbances. While it may be reasonably assumed that a diffuse musculoskeletal disorder or systemic bone frailty such as XLH contributes to widespread pain, its occurrence in localized conditions like monostotic fibrous dysplasia remains unexpected and raises questions about the involvement of additional non-skeletal factors in the pain experience.

In addition, a similar pattern has been observed in adults with other rheumatic conditions. A high prevalence of fibromyalgia, ranging from 10% to 48%, has been reported in adults with rheumatoid arthritis (RA) [36, 42, 43], suggesting that nociplastic pain may represent an intrinsic component of rheumatic diseases rather than a comorbidity. Furthermore, widespread pain, as measured by the WPI, has been identified as a predictor of disease activity in RA [42, 43] and patients experiencing nociplastic features also tend to report worse patient-reported outcomes, including greater fatigue and mood disturbances [42].

Ultimately, our study highlights the importance of looking beyond bone pathology for pain management, and emphasizes the need for careful assessment for the potential aetiology of pain to inform integrative multimodal strategies and interventions targeting central components of pain (e.g. psychological therapies, rehabilitative approaches and anti-neuropathic medications) [9, 44].

This is the first study to provide a comprehensive analysis of the prevalence and characteristics of pain across three rare bone diseases—FD/MAS, OI, and XLH—yielding novel insights into the pain experience within these populations. Despite the rarity of these conditions, our cohort included a substantial sample size of 281 patients, enabling a reliable characterization of pain patterns and the identification of contributing factors. However, our study has several limitations, including its cross-sectional design, which allows for the description of associations but does not permit conclusions about causality, and may be subject to selection bias. Our study is based on an online registry relying solely on patient self-reported data, without access to medical records. Clinical and biological information are inconsistently reported in RUDY, and the absence of medical supervision further limited its reliability. Consequently, analyses were restricted to basic demographic parameters (e.g., age and sex) and did not allow correlation of pain with patients’ disease status (e.g., fractures, hypophosphatemia) or relevant interventions (e.g., surgery, medications), which are known to influence pain trajectories. Another limitation of online registries is their reliance on voluntary participation, which may induce selection bias toward patients with higher health literacy or more severe conditions, potentially leading to an overestimation of pain within our cohort. Digital access can also introduce selection bias, particularly among older individuals who may have limited technological proficiency. Despite these limitations, the stability of pain-related parameters across the three disease groups, including pain phenotype, suggests that pain assessment in our study remains reliable and consistent. In this cross-sectional study, various questionnaires (PD-Q, HADS, PQSI, etc.) were assessed at baseline. Since pain is a dynamic parameter subject to change over time, it would be valuable to investigate its progression in a longitudinal study, examining the impact of treatment as well as other key determinants of pain, such as age. We were unable to accurately assess the pelvic area, as the PD-Q body map did not provide sufficient regional specificity. As described in the methods section, the WPI was determined using the PQ-Q location mapping, with slight modifications in labelling for 2 of the 19 areas. Nevertheless, it is unlikely that these small differences significantly influence the results, given that the same regions are covered. This exploratory study was not specifically designed to meet fibromyalgia diagnosis criteria, and as such, may contain inherent biases. Nevertheless, this preliminary work underscores the urgent need to assess nociplastic pain and fibromyalgia in patients with rare bone diseases.

## Conclusion

Despite distinct pathophysiological mechanisms, the prevalence and main characteristics of pain appear similar across these bone diseases, suggesting that non-bone-related factors may contribute significantly. Pain sites appear to be specific to the underlying bone disease, while female sex and certain pain phenotypes (neuropathic-like and unclear) may represent important determinants of pain.

The common observation of generalized pain along its associations with anxiety, depression, and sleep disturbances in these three rare bone diseases suggest nociplastic features, but requires further investigation.

## Supplementary Information

Below is the link to the electronic supplementary material.

Supplementary Material 1

## Data Availability

Data availability is restricted due to licensing agreements for RUDY study and is therefore not publicly accessible. However, anonymized data can be accessed upon reasonable request through the researcher section of the RUDY website (www.rudystudy.org), and with permission of RUDY.

## Abbreviations

FD/MAS: Fibrous Dysplasia/McCune-Albright Syndrome.
HADS: Hospital Anxiety and Depression Scale
MAS: McCune-Albright Syndrome
OI: Osteogenesis Imperfecta
PD-Q: PainDETECT Questionnaire
PSQI: Pittsburgh Sleep Quality Index
RUDY: Rare and Undiagnosed Diseases Study
WPI: Widespread Pain Index
XLH: X-linked Hypophosphataemia

## References

[1] Pickering ME, Delay M, Morel V. Chronic pain and Bone-Related pathologies: A narrative review. J Pain Res. 2024;17:2937–47. 39253740 10.2147/JPR.S469229 PMC11382656

[2] Hawker GA, Gignac MA, Badley E, Davis AM, French MR, Li Y, et al. A longitudinal study to explain the pain-depression link in older adults with osteoarthritis. Arthritis Care Res (Hoboken). 2011;63(10):1382–90. 20662042 10.1002/acr.20298

[3] Gómez Penedo JM, Rubel JA, Blättler L, Schmidt SJ, Stewart J, Egloff N, et al. The complex interplay of Pain, Depression, and anxiety symptoms in patients with chronic pain: A network approach. Clin J Pain. 2020;36(4):249–59. 31899722 10.1097/AJP.0000000000000797

[4] Mantyh PW. Mechanisms that drive bone pain across the lifespan. Br J Clin Pharmacol. 2019;85(6):1103–13. 30357885 10.1111/bcp.13801 PMC6533434

[5] Oostinga D, Steverink JG, van Wijck AJM, Verlaan JJ. An Understanding of bone pain: A narrative review. Bone. 2020;134:115272. 32062002 10.1016/j.bone.2020.115272

[6] Freynhagen R, Baron R, Gockel U, Tölle TR. PainDETECT: a new screening questionnaire to identify neuropathic components in patients with back pain. Curr Med Res Opin. 2006;22(10):1911–20. 17022849 10.1185/030079906X132488

[7] Hochman JR, Gagliese L, Davis AM, Hawker GA. Neuropathic pain symptoms in a community knee OA cohort. Osteoarthritis Cartilage. 2011;19(6):647–54. 21440077 10.1016/j.joca.2011.03.007

[8] Bittencourt JV, Bezerra MC, Pina MR, Reis FJJ, de Sá Ferreira A, Nogueira LAC. Use of the paindetect to discriminate musculoskeletal pain phenotypes. Arch Physiother. 2022;12(1):7. 35172904 10.1186/s40945-022-00129-2 PMC8851806

[9] Spencer TL, Watts L, Soni A, Pinedo-Villanueva R, Heegaard AM, Boyce AM, et al. Neuropathic-like pain in fibrous Dysplasia/McCune-Albright syndrome. J Clin Endocrinol Metab. 2022;107(6):e2258–66. 35262711 10.1210/clinem/dgac120 PMC9113795

[10] Muñoz Cortés R, Soriano Pastor JF, Monsalve Dolz V. Chronic pain in adults with osteogenesis imperfecta and its relationship to appraisal, coping, and quality of life: A cross-sectional study. Med (Baltim). 2022;101(40):e30256. 10.1097/MD.0000000000030256 PMC9542837 36221335

[11] Cole S, Sanchez-Santos MT, Kolovos S, Javaid MK, Pinedo-Villanueva R. Patient-reported outcomes measures of X-linked hypophosphataemia participants: findings from a prospective cohort study in the UK. Orphanet J Rare Dis. 2023;18(1):26. 36755338 10.1186/s13023-023-02620-w PMC9906829

[12] Diaz-delCastillo M, Espersen RB, Beck-Nielsen SS, Rejnmark L, Heegaard AM, Pain. Quality of life and mental health in adults with X-linked hypophosphatemia: a cross-sectional study. J Clin Endocrinol Metab. 2025. 10.1210/clinem/dgaf104 39969548

[13] Wolfe F, Clauw DJ, Fitzcharles MA, Goldenberg DL, Häuser W, Katz RL, et al. 2016 revisions to the 2010/2011 fibromyalgia diagnostic criteria. Semin Arthritis Rheum. 2016;46(3):319–29. 27916278 10.1016/j.semarthrit.2016.08.012

[14] Zigmond AS, Snaith RP. The hospital anxiety and depression scale. Acta Psychiatr Scand. 1983;67(6):361–70. 6880820 10.1111/j.1600-0447.1983.tb09716.x

[15] White D, Leach C, Sims R, Atkinson M, Cottrell D. Validation of the hospital anxiety and depression scale for use with adolescents. Br J Psychiatry. 1999;175:452–4. 10789277 10.1192/bjp.175.5.452

[16] Mollayeva T, Thurairajah P, Burton K, Mollayeva S, Shapiro CM, Colantonio A. The Pittsburgh sleep quality index as a screening tool for sleep dysfunction in clinical and non-clinical samples: A systematic review and meta-analysis. Sleep Med Rev. 2016;25:52–73. 26163057 10.1016/j.smrv.2015.01.009

[17] Buysse DJ, Reynolds CF 3rd, Monk TH, Berman SR, Kupfer DJ. The Pittsburgh sleep quality index: a new instrument for psychiatric practice and research. Psychiatry Res. 1989;28(2):193–213. 10.1016/0165-1781(89)90047-4 2748771

[18] Majoor BCJ, Traunmueller E, Maurer-Ertl W, Appelman-Dijkstra NM, Fink A, Liegl B, et al. Pain in fibrous dysplasia: relationship with anatomical and clinical features. Acta Orthop. 2019;90(4):401–5. 31035847 10.1080/17453674.2019.1608117 PMC6718189

[19] Osborne NR, Davis KD. Sex and gender differences in pain. Int Rev Neurobiol. 2022;164:277–307. 36038207 10.1016/bs.irn.2022.06.013

[20] Pieretti S, Di Giannuario A, Di Giovannandrea R, Marzoli F, Piccaro G, Minosi P, et al. Gender differences in pain and its relief. Ann Ist Super Sanita. 2016;52(2):184–9. 27364392 10.4415/ANN_16_02_09

[21] Hallin RG. [Pain more painful in women. Gender perspective neglected in research on the biological mechanisms of pain]. Lakartidningen. 2003;100(46):3738–41. 14655329

[22] Rodriguez Celin M, Kruger KM, Caudill A, Murali CN, Nagamani SCS, Smith PA, et al. A multicenter study to evaluate pain characteristics in osteogenesis imperfecta. Am J Med Genet A. 2023;191(1):160–72. 36271817 10.1002/ajmg.a.63009 PMC10399129

[23] Murray CB, Patel KV, Twiddy H, Sturgeon JA, Palermo TM. Age differences in cognitive-affective processes in adults with chronic pain. Eur J Pain. 2021;25(5):1041–52. 33405280 10.1002/ejp.1725 PMC8055045

[24] Lautenbacher S, Peters JH, Heesen M, Scheel J, Kunz M. Age changes in pain perception: A systematic-review and meta-analysis of age effects on pain and tolerance thresholds. Neurosci Biobehav Rev. 2017;75:104–13. 28159611 10.1016/j.neubiorev.2017.01.039

[25] El Tumi H, Johnson MI, Dantas PBF, Maynard MJ, Tashani OA. Age-related changes in pain sensitivity in healthy humans: A systematic review with meta-analysis. Eur J Pain. 2017;21(6):955–64. 28230292 10.1002/ejp.1011

[26] Kelly MH, Brillante B, Collins MT. Pain in fibrous dysplasia of bone: age-related changes and the anatomical distribution of skeletal lesions. Osteoporos Int. 2008;19(1):57–63. 17622477 10.1007/s00198-007-0425-x

[27] Florenzano P, Pan KS, Brown SM, Paul SM, Kushner H, Guthrie LC, et al. Age-Related changes and effects of bisphosphonates on bone turnover and disease progression in fibrous dysplasia of bone. J Bone Min Res. 2019;34(4):653–60. 10.1002/jbmr.3649 PMC6983318 30645769

[28] Skrinar A, Dvorak-Ewell M, Evins A, Macica C, Linglart A, Imel EA, et al. The lifelong impact of X-Linked hypophosphatemia: results from a burden of disease survey. J Endocr Soc. 2019;3(7):1321–34. 31259293 10.1210/js.2018-00365 PMC6595532

[29] Cheung M, Rylands AJ, Williams A, Bailey K, Bubbear J. Patient-Reported Complications, Symptoms, and experiences of living with X-Linked hypophosphatemia across the Life-Course. J Endocr Soc. 2021;5(8):bvab070. 34258488 10.1210/jendso/bvab070 PMC8272533

[30] Ito N, Kang HG, Nishida Y, Evins A, Skrinar A, Cheong HI. Burden of disease of X-linked hypophosphatemia in Japanese and Korean patients: a cross-sectional survey. Endocr J. 2022;69(4):373–83. 34732603 10.1507/endocrj.EJ21-0386

[31] Namba N, Ito N, Michigami T, Kang HG, Kubota T, Miyazaki O, et al. Impact of X-linked hypophosphatemic rickets/osteomalacia on health and quality of life: baseline data from the SUNFLOWER longitudinal, observational cohort study. JBMR Plus. 2024;8(11):ziae118. 39399158 10.1093/jbmrpl/ziae118 PMC11470975

[32] Barlow S, Dove L, Jaggi A, Keen R, Bubbear J. The prevalence of musculoskeletal pain and therapy needs in adults with osteogenesis imperfecta (OI) a cross-sectional analysis. BMC Musculoskelet Disord. 2022;23(1):485. 35598006 10.1186/s12891-022-05433-3 PMC9123157

[33] Lo SH, Lachmann R, Williams A, Piglowska N, Lloyd AJ. Exploring the burden of X-linked hypophosphatemia: a European multi-country qualitative study. Qual Life Res. 2020;29(7):1883–93. 32162120 10.1007/s11136-020-02465-x PMC7295835

[34] Golden E, van der Heijden H, Ren B, Randall ET, Drubach LA, Shah N, et al. Phenotyping pain in patients with fibrous Dysplasia/McCune-Albright syndrome. J Clin Endocrinol Metab. 2024;109(3):771–82. 37804088 10.1210/clinem/dgad589 PMC11491648

[35] Haliloglu S, Carlioglu A, Akdeniz D, Karaaslan Y, Kosar A. Fibromyalgia in patients with other rheumatic diseases: prevalence and relationship with disease activity. Rheumatol Int. 2014;34(9):1275–80. 24589726 10.1007/s00296-014-2972-8

[36] Murphy AE, Minhas D, Clauw DJ, Lee YC. Identifying and managing nociplastic pain in individuals with rheumatic diseases: A narrative review. Arthritis Care Res (Hoboken). 2023;75(10):2215–22. 36785994 10.1002/acr.25104 PMC11210328

[37] Kaplan CM, Kelleher E, Irani A, Schrepf A, Clauw DJ, Harte SE. Deciphering nociplastic pain: clinical features, risk factors and potential mechanisms. Nat Rev Neurol. 2024;20(6):347–63. 38755449 10.1038/s41582-024-00966-8

[38] Bułdyś K, Górnicki T, Kałka D, Szuster E, Biernikiewicz M, Markuszewski L et al. What do we know about nociplastic pain? Healthc (Basel). 2023;11(12). 10.3390/healthcare11121794 PMC10298569 37372912

[39] Hochman JR, Davis AM, Elkayam J, Gagliese L, Hawker GA. Neuropathic pain symptoms on the modified paindetect correlate with signs of central sensitization in knee osteoarthritis. Osteoarthritis Cartilage. 2013;21(9):1236–42. 23973136 10.1016/j.joca.2013.06.023

[40] Soni A, Wanigasekera V, Mezue M, Cooper C, Javaid MK, Price AJ, et al. Central sensitization in knee osteoarthritis: relating presurgical brainstem neuroimaging and PainDETECT-Based patient stratification to arthroplasty outcome. Arthritis Rheumatol. 2019;71(4):550–60. 30295432 10.1002/art.40749 PMC6430421

[41] Palmisano B, Tavanti C, Farinacci G, Gosti G, Leonetti M, Donsante S et al. Bone pain in fibrous dysplasia does not rely on aberrant sensory nerve sprouting or neuroma formation. J Bone Min Res. 2025. 10.1093/jbmr/zjaf066 PMC12308826 40324210

[42] Chaabo K, Chan E, Garrood T, Rutter-Locher Z, Vincent A, Galloway J et al. Pain sensitisation and joint inflammation in patients with active rheumatoid arthritis. RMD Open. 2024;10(1). 10.1136/rmdopen-2023-003784 PMC10953307 38508678

[43] Kozanoğlu E, Kelle B, Alaylı G, Kuru Ö, Çubukçu Fırat S, Demir AN, et al. Frequency of fibromyalgianess in patients with rheumatoid arthritis and ankylosing spondylitis: A multicenter study of Turkish league against rheumatism (TLAR) network. Arch Rheumatol. 2024;39(1):20–32. 38774695 10.46497/ArchRheumatol.2023.9925 PMC11104752

[44] Javaid MK, Boyce A, Appelman-Dijkstra N, Ong J, Defabianis P, Offiah A, et al. Best practice management guidelines for fibrous dysplasia/McCune-Albright syndrome: a consensus statement from the FD/MAS international consortium. Orphanet J Rare Dis. 2019;14(1):139. 31196103 10.1186/s13023-019-1102-9 PMC6567644

