# Supplementary material for: Addressing the unmet challenge of pain in rare bone diseases: new insights from the RUDY UK registry
Source: Orphanet J Rare Dis. 2026 Jan 29;21:33. doi: 10.1186/s13023-025-04167-4 (PMC12857081; doi:10.1186/s13023-025-04167-4)
Supplement: Supplementary file 1 — Supplementary Material 1 [file 13023_2025_4167_MOESM1_ESM.docx]

**Supplemental figures**

**Supplemental figure 1:** PainDETECT questionnaire (online version from https://test.rudystudy.org/)


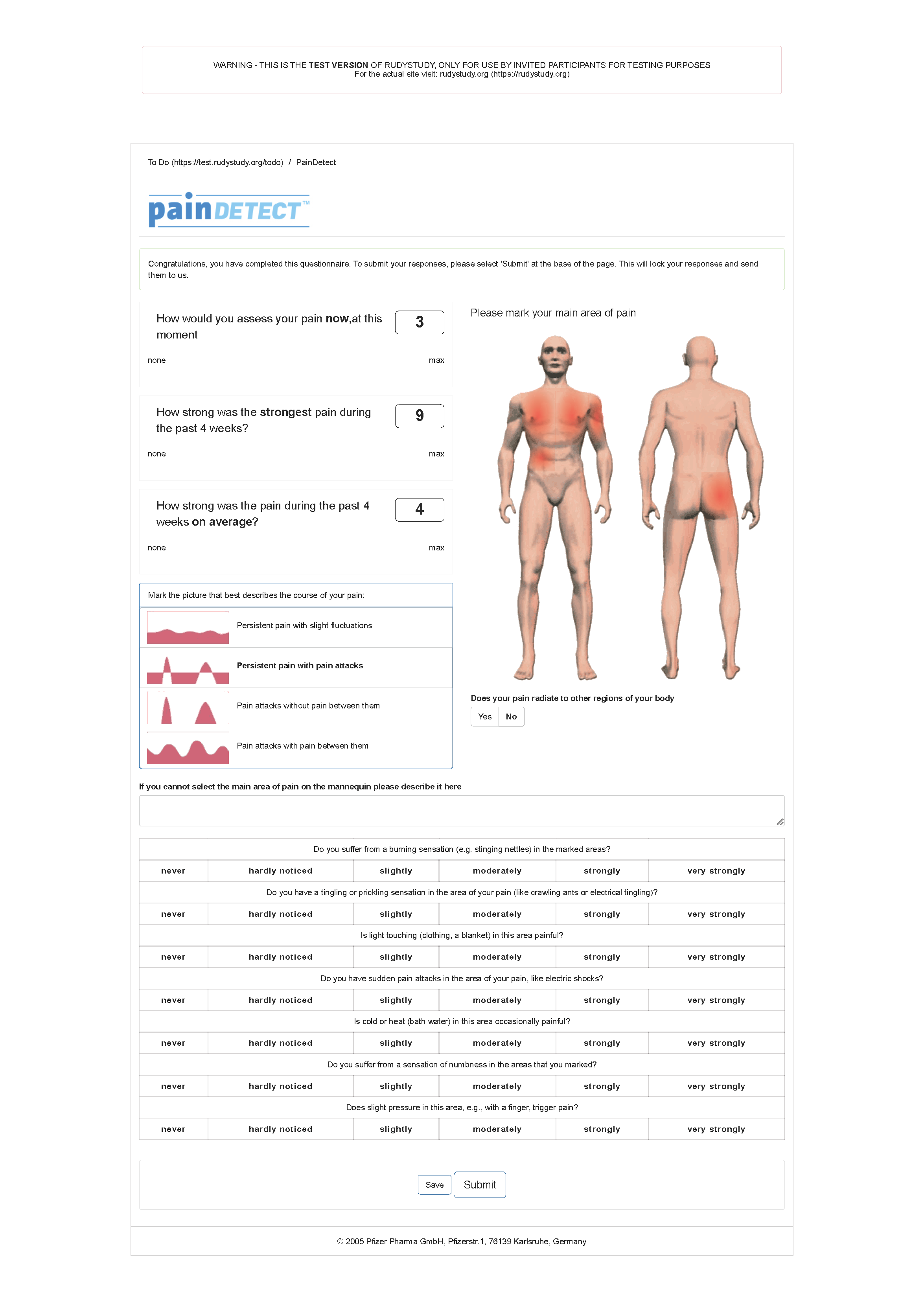


**Supplemental tables**

**Supplemental table 1.** Definition of Generalized Pain and Corresponding Body Regions According to WPI and Modified WPI

|  | **Region 1 :**  **Left upper region** | **Region 2 :**  **Right upper region** | **Region 3 :**  **Left lower region** | **Region 4 :**  **Right lower region** | **Region 5 :**  **Axial region** |
| --- | --- | --- | --- | --- | --- |
| **WPI** | Jaw (L)  Shoulder girdle (L)  Upper arm (L)  Lower arm (L) | Jaw (R)  Shoulder girdle (R)  Upper arm (R)  Lower arm (R) | Hip (L)  Upper leg (L)  Lower leg (L) | Hip (R)  Upper leg (R)  Lower leg (R) | Neck  Upper back*  Lower back*  Chest/breast  Abdomen |
| **Modified-WPI** | Jaw (L)  Shoulder (L)  Upper arm (L)  Lower arm (L) | Jaw (R)  Shoulder (R)  Upper arm (R)  Lower arm (R) | Hip (L)  Upper leg (L)  Lower leg (L) | Hip (R)  Upper leg (R)  Lower leg (R) | Neck  Back*  Spine*  Chest  Abdomen |
| **Generalized pain** | **Pain in at least 4 ouf 5 regions.**  Jaw, chest and abdominal pain are not included in generalized pain definition. | | | | |

*Comparison of WPI vs.  « Modified WPI ». Body regions and generalized pain were defined according to the 2016 ACR criteria for fibromyalgia (Wolfe F et al., Semin Arthritis Rheum, 2016).*

**The axial region was slightly modified in this equivalent WPI because the back areas were labeled and divided differently (lower/upper back in WPI vs. back and spine in modified WPI), although the entire back/spine region was similarly covered.*

*L: left; R: right.*

**Supplemental table 2.** Subgroup analysis in adults with FD/MAS

**2a.** Pain characteristics according to gender

| **FD/MAS** | **All** | **Female** | **Male** | **P** |
| --- | --- | --- | --- | --- |
| **Patients characteristics** |  |  |  |  |
| Total subjects | 94 | 67(71%) | 27(29%) |  |
| Median age, years (range) | 47(18-74) | 44(18-74) | 53(18-69) |  |
| Women |  |  |  |  |
| FD type |  |  |  |  |
| monostotic | 27(29%) | 19(28%) | 8(30%) |  |
| polyostotic | 32(34%) | 23(34%) | 9(33%) |  |
| no FD type avalaible | 35(37%) | 25(37%) | 10(37%) |  |
| MAS | 15(16%) |  |  |  |
| Cranio-facial FD | 24(26%) |  |  |  |
| **Pain intensity (0-10)** |  |  |  |  |
| Current pain | 4.5(1.3-7) | 5 (3-7) | 2(1-7) | 0.31 |
| Average pain in last 4 weeks | 5 (3-7) | 5 (3-7) | 4(1.5-6.5) | 0.21 |
| Strongest pain in last 4 weeks | 8(5-9) | 8(6-9) | 7(3.5-9) | 0.11 |
| **Pain occurrence (y/n)** |  |  |  |  |
| Current pain (y) | 81(86%) | 59(88%) | 22(81%) | 0.51 |
| Strongest pain ≥8/10 in last 4 weeks (y) | 50(53%) | 40(60%) | 10(37%) | 0.08 |
| **Extent of painful location** |  |  |  |  |
| Number of painful areas (1-66) | 3 (2-5.75) | 3(1 ;5-6) | 3(2-5) | 0.90 |
| Modified -WPI generalized pain | 11(12%) | 10 (15%) | 1 (4%) |  |
| **Pain phenotype** |  |  |  | 0.53 |
| nociceptive | 54(60%) | 37(57%) | 17(68%) |  |
| neuropathic-like | 15(17%) | 11(17%) | 4(16%) |  |
| unclear | 21(23%) | 17(26%) | 4(16%) |  |
| missing data | 4(4%) | 2(3%) | 2(7%) |  |
| **Pain location** |  |  |  |  |
| Cranio-facial | 26(28%) | 15(22%) | 11(41%) | 0.12 |
| upper-limb | 34(36%) | 26(39%) | 8(30%) | 0.55 |
| lower-limb | 60(64%) | 45(67%) | 15(56%) | 0.41 |
| axial | 39(42%) | 30(45%) | 9(33%) | 0.43 |
| **Course pain profile** |  |  |  | 0.11 |
| Persistent pain with slight fluctuations | 15(17%) | 12(18%) | 3(12%) |  |
| Persistent pain with pain attacks | 36(40%) | 30(46%) | 6(24%) |  |
| Pain attacks without pain between them | 26(29%) | 15(23%) | 11(44%) |  |
| Pain attacks with pain between them | 13(14%) | 8(12%) | 5(20%) |  |
| Missing datas | 4(4% | 2(3%) | 2(7%) |  |

*Data are expressed as median (IQR) or n (%), unless otherwise indicated. y/n : yes or no ; y : yes.*

**2b.** Association of pain outcomes with age and gender in FD/MAS adults

|  | **Age** | | | **Gender Male** | | |
| --- | --- | --- | --- | --- | --- | --- |
| **Pain occurrence (y/n)** | **OR** | **95% CI** | **p** | **OR** | **95% CI** | **p** |
| Current pain (y) | 1.00 | 0.95 ; 1.04 | 0.84 | 0.61 | 0.18 ; 2.27 | 0.44 |
| Severe strongest pain in past 4 weeks (y) | 0.98 | 0.95 ; 1.01 | 0.17 | 0.45 | 0.17 ; 1.14 | 0.09 |
| Generalized pain (y) | 1.02 | 0.98 ; 1.07 | 0.37 | 0.19 | 0.01 ; 1.11 | 0.13 |
| **Pain intensity (0-10)** | **Coefficients** | **95% CI** | **p** | **Coefficients** | **95% CI** | **p** |
| Current pain | -0.02 | -0.06 ; 0.02 | 0.34 | -0.52 | -1.90 ; 0.86 | 0.46 |
| Average pain in past 4 weeks | -0.02 | -0.06 ; 0.02 | 0.23 | -0.68 | -1.94 ; 0.58 | 0.29 |
| Strongest pain in past 4 weeks | -0.02 | -0.06 ; 0.02 | 0.27 | -1.25 | -2.55 ; 0.05 | 0.06 |
| **Extent of painful location** | **Coefficients** | **95% CI** | **p** | **Coefficients** | **95% CI** | **p** |
| Number of painful areas (1-66) | 0.02 | -0.07 ; 0.11 | 0.60 | -2.04 | -4.92 ; 0.83 | 0.16 |
| **Pain phenotype** | **OR** | **95% CI** |  | **OR** | **95% CI** |  |
| neuropathic | 0.96 | 0.92 ; 1.00 |  | 1.05 | 0.27 ; 4.03 |  |
| unclear | 1.00 | 0.96 ; 1.04 |  | 0.52 | 0.15 ; 1.83 |  |

*Results, derived from linear or logistic regression analyses, are presented as coefficients or Odds Ratios (ORs) with corresponding 95% confidence intervals (95% CI). Females were used as the reference group. y/n : yes or no ; y : yes.*

**2c.** Pain characteristics based on monostotic or polyostotic FD status

| **FD/MAS** | **All** | **Mono** | **Poly** | **p** |
| --- | --- | --- | --- | --- |
| **Patients characteristics** |  |  |  |  |
| Total subjects | 94 | 27 | 32 |  |
| Median age, years (range) | 47(18-74) | 48 (18-71) | 47(19-66) | 0.75 |
| Women |  | 19 | 23 | 1 |
| FD type |  |  |  |  |
| monostotic | 27(29%) |  |  |  |
| polyostotic | 32(34%) |  |  |  |
| no FD type avalaible | 35(37%) |  |  |  |
| MAS | 15(16%) |  |  |  |
| Cranio-facial FD | 24(26%) |  |  |  |
| **Pain intensity (0-10)** |  |  |  |  |
| Current pain | 4.5(1.3-7) | 3(1-6.5) | 5(3-7) | 0.2 |
| Average pain in last 4 weeks | 5 (3-7) | 5(1-6.5) | 5(4-7) | 0.26 |
| Strongest pain in last 4 weeks | 8(5-9) | 7(3-9) | 7.5(6-9) | 0.61 |
| **Pain occurrence (y/n)** |  |  |  |  |
| Current pain (y) | 81(86%) | 24(89%) | 31(97%) | 0.32 |
| Strongest pain ≥8/10 in last 4 weeks (y) | 50(53%) | 13(48%) | 16(50%) | 1 |
| **Extent of painful location** |  |  |  |  |
| Number of painful areas (1-66) | 3 (2-5.75) | 2(1-2.5) | 4.5(2-6,25) | <0.01* |
| Modified -WPI generalized pain | 11(11,70%) | 2(7%) | 4(13%) | 0.68 |
| **Pain phenotype** |  |  |  | 0.08 |
| Nociceptive | 54(60%) | 15(63%) | 19(59%) |  |
| Neuropathic-like | 15(17%) | 2(8%) | 8(25%) |  |
| Unclear | 21(23%) | 7(29%) | 5(16%) |  |
| Missing data | 4(4%) | 3(11%) | 0(0%) |  |
| **Pain location** |  |  |  |  |
| Cranio-facial | 26(28%) | 9(33%) | 5(16%) | 0.13 |
| Upper-limb | 34(36%) | 9(33%) | 11(34%) | 1 |
| Lower-limb | 60(64%) | 16(59%) | 24(75%) | 0.31 |
| Axial | 39(42%) | 9(33%) | 16(50%) | 0.3 |
| **Course pain profile** |  |  |  | 0.18 |
| Persistent pain with slight fluctuations | 15(17%) | 4(17%) | 6(19%) |  |
| Persistent pain with pain attacks | 36(40%) | 8(33%) | 16(50%) |  |
| Pain attacks without pain between them | 26(29%) | 10(42%) | 7(22%) |  |
| Pain attacks with pain between them | 13(14%) | 2(8%) | 3(9%) |  |
| Missing datas | 4(4% | 3(11%) | 0(0%) |  |

*Data are expressed as median (IQR) or n (%), unless otherwise indicated. y/n : yes or no ; y : yes.*

**Supplemental table 3.** Subgroup analysis in adults with OI

**3a.** Pain characteristics in adults with OI according to gender

| **OI** | **All** | **Female** | **Male** | **P** |
| --- | --- | --- | --- | --- |
| **Patients characteristics** |  |  |  |  |
| Total subjects | 94 | 69(73%) | 25(36%) |  |
| Median age, years (range) | 43(21-75) | 43 (21-74) | 45 (21 -78) | 0.39 |
| OI subtype |  |  |  |  |
| - type 1 | 32(34%) |  |  |  |
| - type 3 | 8(9%) |  |  |  |
| - type 4 | 8(9%) |  |  |  |
| - type 5 | 1(1%) |  |  |  |
| - undetermined | 45(48%) |  |  |  |
| **Pain intensity (0-10)** |  |  |  |  |
| Current pain | 3(1-5) | 4 (1-6) | 2(1-4) | 0.05* |
| Average pain in last 4 weeks | 4(3-6) | 5 (3-6) | 3(2-5) | 0.04* |
| Strongest pain in last 4 weeks | 6(4-8) | 7(5-8) | 6(4-7) | 0.03* |
| **Pain occurrence (y/n)** |  |  |  |  |
| Current pain (y) | 82(87%) | 62 (90%) | 20 (80%) | 0.29 |
| Strongest pain ≥8/10 in last 4 weeks (y) | 35(37%) | 31 (45%) | 4 (16%) | 0.01* |
| **Extent of painful location** |  |  |  |  |
| Number of painful areas (1-66) | 7 (4-14) | 9 (4-15) | 5(4-8) | 0.14 |
| Modified-WPI generalized pain | 20(21,28%) | 16 | 4 | 0.57 |
| **Pain phenotype** |  |  |  | 0.02* |
| Neuropathic | 11(12%) | 11(16%) | 0(0%) |  |
| Nociceptive | 63(70%) | 46(68%) | 17(77%) |  |
| Unclear | 16(18%) | 11(16%) | 5(23%) |  |
| Missing data | 4(4%) | 1(1%) | 3(12%) |  |
| **Pain location** |  |  |  |  |
| Cranio-facial | 8(9%) | 6(9%) | 2(8%) | 1 |
| Upper-limb | 49(52%) | 40(58%) | 9(36%) | 0.10 |
| Lower-limb | 83(88%) | 60(87%) | 23(92%) | 0.72 |
| Axial | 71(76%) | 53(77%) | 18(72%) | 0.84 |
| **Course pain profile** |  |  |  | 0.03* |
| Persistent pain with slight fluctuations | 26(29%) | 23(34%) | 3(14%) |  |
| Persistent pain with pain attacks | 19(21%) | 14(21%) | 5(23%) |  |
| Pain attacks without pain between them | 28(31%) | 17(25%) | 11(50%) |  |
| Pain attacks with pain between them | 17(19%) | 14 (21%) | 3(14%) |  |
| Missing datas | 4(4%) | 1(1%) | 3(12%) |  |

Data are expressed as median (IQR) or n (%), unless otherwise indicated. *y/n : yes or no ; y : yes.*

**3b.** Association of pain outcomes with age and gender in adults with OI

|  | **Age** | | | | **Gender M** | | | |
| --- | --- | --- | --- | --- | --- | --- | --- | --- |
| **Pain occurrence (y/n)** | **OR** | **95% CI** | **p** | **OR** | | **95% CI** | **p** |  |
| Current pain (y) | 0.99 | 0.95 ; 1.03 | 0.66 | 0.47 | | 0.13 ; 1.75 | 0.24 |  |
| Severe strongest pain in past 4 weeks (y) | 0.99 | 0.96 ; 1.02 | 0.40 | 0.24 | | 0.06 ; 0.72 | 0.02* |  |
| Generalized pain (y) | 0.97 | 0.94 ; 1.01 | 0.12 | 0.67 | | 0.17 ; 2.13 | 0.52 |  |
| **Pain intensity (0-10)** | **Coefficients** | **95% CI** | **p** | **Coefficients** | | **95% CI** | **p** |  |
| Current pain intensity | -0.02 | -0.06 ; 0.01 | 0.12 | -1.11 | | -2.21 ; -0.003 | 0.05* |  |
| Average pain in past 4 weeks | -0.02 | -0.04 ; 0.01 | 0.29 | -1.03 | | -2.04 ; -0.02 | 0.05* |  |
| Strongest pain in past 4 weeks | -0.02 | -0.05 ; 0.02 | 0.32 | -1.08 | | -2.20 ; 0.05 | 0.06 |  |
| **Extent of painful location** | **Coefficients** | **95% CI** | **p** | **Coefficients** | | **95% CI** | **p** |  |
| Number of painful areas (1-66) | -0.08 | -0.19 ; 0.04 | 0.17 | -2.89 | | -6.88; 1.11 | 0.16 |  |
| **Pain phenotype** | **OR** | **95% CI** |  | | | | |  |
| Neuropathic | 1.01 | 0.96; 1.05 |  |  |  |  |  |  |
| Unclear | 0.99 | 0.96; 1.03 |  |  |  |  |  |  |

*y/n : yes or no ; y : yes.*

**Supplemental table 4.** Subgroup analysis in adults with XLH

**4a.** Pain characteristics in adults with XLH according to gender

| **XLH** | **All** | **Female** | **Male** | **P** |
| --- | --- | --- | --- | --- |
| **Patients characteristics** |  |  |  |  |
| Total subjects | 93 | 69(74%) | 24(26%) |  |
| Median age, years (range) | 41(19-78) | 41 (19-78) | 37 (20-78) | 0.28 |
| **Pain intensity (0-10)** |  |  |  |  |
| Current pain | 4(2-7) | 4 (2-6) | 2(0-7) | 0.13 |
| Average pain in last 4 weeks | 5(3-7) | 5(4-7) | 4(1-7) | 0.15 |
| Strongest pain in last 4 weeks | 8(5-9) | 8(6-9) | 5.5(2-9) | 0.11 |
| **Pain occurrence (y/n)** |  |  |  |  |
| Current pain (y) | 80(86%) | 63 (91%) | 17(71%) | 0.03* |
| Strongest pain ≥8/10 in last 4 weeks (y) | 47(51%) | 37(54%) | 10(42%) | 0.44 |
| **Extent of painful location** |  |  |  |  |
| Number of painful areas (1-66) | 9(4.25  -15.75 | 9(4.5- 15) | 8(5-16) | 0.87 |
| Modified-WPI generalized pain | 20(22%) | 15 (22%) | 5(21%) | 1 |
| **Pain phenotype** |  |  |  | 0.09 |
| Nociceptive | 55(62%) | 40(59%) | 15 (71%) |  |
| Neuropathic-like | 12(13%) | 8(12%) | 4 (19%) |  |
| Uncategorized | 22(25%) | 20(29%) | 2 (10%) |  |
| Missing data | 4(4%) | 1(1%) | 3 (13%) |  |
| **Pain location** |  |  |  |  |
| Cranio-facial | 7 (8%) | 5 (7%) | 2 (10%) | 1 |
| Upper-limb | 43 (48%) | 34 (50%) | 9 (43%) | 0.45 |
| Lower-limb | 87 (98%) | 65 (96%) | 22 (105%) | 0.65 |
| Axial | 51 (57%) | 37 (54%) | 14 (67%) | 0.87 |
| **Course pain profile** |  |  |  | 0.09 |
| Persistent pain with slight fluctuations | 20 (22%) | 13 (19%) | 7 (33%) |  |
| Persistent pain with pain attacks | 30 (34%) | 25 (37%) | 5 (24%) |  |
| Pain attacks without pain between them | 20 (22%) | 14 (21%) | 6 (29%) |  |
| Pain attacks with pain between them | 19 (21%) | 16 (24%) | 3 (14%) |  |
| Missing datas | 4 (4%) | 1 (1%) | 3 (13%) |  |

*Data are expressed as median (IQR) or n (%), unless otherwise indicated. y/n : yes or no ; y : yes.*

**4b.** Association of pain outcomes with age and gender in XLH adults

|  | **Age** | | | **Gender M** | | |
| --- | --- | --- | --- | --- | --- | --- |
| **Pain occurrence (y/n)** | **OR** | **95% CI** | **p** | **OR** | **95% CI** | **p** |
| Current pain (y) | 0.98 | 0.94 ; 1.03 | 0.41 | 0.21 | 0.06 ; 0.73 | 0.01* |
| Severe strongest pain in past 4 weeks (y) | 1.01 | 0.99 ; 1.04 | 0.32 | 0.65 | 0.25 ; 1.68 | 0.38 |
| Generalized pain(y) | 1.02 | 0.99 ; 1.06 | 0.15 | 1.06 | 0.31 ; 3.26 | 0.92 |
| **Pain intensity (0-10)** | **Coefficients** | **95% CI** | **p** | **Coefficients** | **95% CI** | **p** |
| Current pain | 0.003 | -0.037 ; 0.043 | 0.89 | -0.970 | -2.320 ; 0.379 | 0.16 |
| Average pain in past 4 weeks | 0.001 | -0.036 ; 0.037 | 0.98 | -1.003 | -2.226 ; 0.220 | 0.11 |
| Strongest pain in past 4 weeks | -0.003 | -0.041 ; 0.035 | 0.89 | -1.527 | -2.812 ; -0.243 | 0.02* |
| **Extent of painful location** | **Coefficients** | **95% CI** | **p** | **Coefficients** | **95% CI** | **p** |
| Number of painful areas (1-66) | 0.01 | -0.12 ; 0.14 | 0.89 | 1.32 | -2.801 ; 5.73 | 0.50 |
| **Pain phenotype** | **OR** | **95% CI** |  | **OR** | **95% CI** |  |
| neuropathic | 1.03 | 0.98 ; 1.07 |  | 1.40 | 0.36 ; 5.46 |  |
| unclear | 1.02 | 0.99 ; 1.06 |  | 0.28 | 0.06 ; 1.35 |  |

*p<0.05 ; *y/n : yes or no ; y : yes.*
